# The aspect of structural connectivity in relation to age-related gait performance

**DOI:** 10.1093/psyrad/kkad028

**Published:** 2023-11-27

**Authors:** Cheol-Woon Kim, Yechan Kim, Hyun-Ho Kim, Joon Yul Choi

**Affiliations:** Department of Physical Education, Korea University,, 02841, Seoul, Republic of Korea; Department of Biomedical Engineering, Yonsei University,, 26493, Wonju, Republic of Korea; Department of Biomedical Engineering, Yonsei University,, 26493, Wonju, Republic of Korea; Department of Biomedical Engineering, Yonsei University,, 26493, Wonju, Republic of Korea

Fernandez *et al*. (2019). investigated the relationship between age-related changes in attention control and gait performance in older adults. The study aimed to establish a more precise link between the influence of age on brain systems mediating executive cognitive functions and their relationship with gait disturbances. Event-related functional magnetic resonance imaging (fMRI) data were acquired from 20 individuals (21 ± 3 years) and 34 older adults (72 ± 5 years) with no history of neurological, psychiatric, or toxicological diseases to evaluate age-related effects on the activation of the executive control brain network during a selective attention task. The study compared brain activation patterns between young and older participants with fMRI, and examined an association between age-related differences in activation patterns and gait parameters, cognitive abilities, and physical abilities.

The study demonstrated a greater sensitivity to attention interference and heightened recruitment of cortical executive control systems in elderly individuals with poor gate performance. These findings were associated with selective increases in gait variability indices. The older participants exhibited differential recruitment of the left dorsal parieto-occipital sulcus and precuneus, which were significantly correlated with higher gait variability. This suggests a connection between attentional control and gait performance in older individuals.

## Neuroplasticity

The ability to make adaptive changes related to the structural and function of the nervous system, known as neuroplasticity, has become an important aspect of neuroscience (May, 2011). Numerous clinical and neuroscience studies have provided substantial evidence to support neuroplasticity.

Rehabilitation has enhanced the brain function of adult stroke patients (over 18 years old) who experienced disability following a cerebrovascular event, promoting structural changes in both gray matter (motor areas) and white matter (Gauthier *et al*., 2008; Diao *et al*., 2017). The group (average age 44 ± 15 years) that received 12 weeks of balance training showed a significant improvement in balance performance, along with an increase in cortical thickness in various brain regions associated with visual and vestibular self-motion perception (Rogge *et al*., 2018). Another study (average age 68 ± 6 years) also demonstrated improved memory performance, accompanied by an increase in the size of the hippocampus after 1 year of aerobic exercise (Erickson *et al*., 2011). The adult group (average age 63 ± 4 years) that underwent 12 weeks of cognitive training revealed improved connectivity in the central executive network and increased white matter integrity in the left uncinate fasciculus (Chapman *et al*., 2015). Training focused on attention resulted in increases in cortical thickness in prefrontal regions, socio-affective training induced increases in frontoinsular regions, and socio-cognitive training altered inferior frontal and lateral temporal cortices in adults (average age 41 ± 9 years) who received mental training for 9 months (Valk *et al*., 2017).

The studies all highlight that the brain continues to change even in adulthood. Although no all-inclusive theory spans different frameworks in the study of neuroplasticity, there are two important biological hypotheses to explain it: functional plasticity and structural plasticity. Functional plasticity describes the brain's capability to execute diverse functions or cooperate with other brain regions to compensate for various functions (Demarin *et al*., 2014). Interestingly, structural changes in the brain, known as structural plasticity, also occur to modify its organization or patterns of connectivity (Butz *et al*., 2009).

Fernandez *et al*. precisely investigated the association between age and gait variability using task-based fMRI in terms of functional plasticity. We hypothesized that there would be age-related differences in structural strength within the executive control network, providing evidence for the functional changes observed in the study.

## Tractography

Diffusion tensor imaging takes anisotropic features of water molecule diffusion within white matter fibers. This imaging modality captures tensor information, which is subsequently utilized to compute eigenvectors and eigenvalues to represent the characteristics of the ellipsoid shape of the fibers (Assaf and Pasternak, 2008). Eigenvectors and eigenvalues are applied to estimate local fiber orientations and trace streamlines, a technique known as tractography. (Nucifora *et al*., 2007; Pal *et al*., 2012). Tractography can be generated based on deterministic and probabilistic approaches. Deterministic tractography does not calculate uncertainty during the fiber orientation estimation process and is relatively fast. On the other hand, probabilistic tractography takes uncertainty into account, but it requires more computational calculations compared to deterministic tractography (Sarwar, 2019). The indices obtained from tractography include the number of streamlines and tract volume. Tractography has been linked to structural connectivity and functional changes (Assaf, 2019; Zhang *et al*., 2022).

In patients with multiple sclerosis, working memory impairment and a decrease in the number of fiber connections were observed (Audoin *et al*., 2007). Quantitative analysis using tractography demonstrated that musicians exhibit heightened motor coordination, as evidenced by increased track volume and the number of streamlines in the superior and middle cerebellar peduncles compared to controls (Snell, 2001; Abdul-Kareem *et al*., 2011). Patients with COVID-19 demonstrated a significant association between olfactory dysfunction and reduced integration as well as increased segregation in olfactory-related brain regions. These findings provide insights into compensatory plasticity mechanisms (Bispo *et al*., 2023).

There is a potential challenge in tractography. Streamlines in tractography are constructed based on their likelihood, and errors can occur during the fiber tracking process (Jeurissen *et al*., 2019). However, it remains valuable for investigating relationships between structural and functional changes in the brain using tractography.

## Conclusion

This study reviewed the evidence for neuroplasticity and introduced the feasibility of using tractography in relating brain plasticity. From the perspective of structural plasticity, we anticipate that older adults may exhibit smaller track volume or fewer streamlines than younger individuals in the executive control networks and compensatory regions such as the precuneus and dorsal parieto-occipital cortices as observed in Fernandez *et al*.'s study. These findings would enhance the reliability of the research and provide additional supporting evidence for the functional changes.
